# Neurological Mechanisms of Animal-Assisted Intervention in Alzheimer’s Disease: A Hypothetical Review

**DOI:** 10.3389/fnagi.2021.682308

**Published:** 2021-07-14

**Authors:** Sujin Kim, Yunkwon Nam, Min-Joo Ham, Chisoo Park, Minho Moon, Doo-Han Yoo

**Affiliations:** ^1^Department of Biochemistry, College of Medicine, Konyang University, Daejeon, South Korea; ^2^Research Institute for Dementia Science, Konyang University, Daejeon, South Korea; ^3^Department of Occupational Therapy, Konyang University, Daejeon, South Korea

**Keywords:** dementia, Alzheimer’s disease, animal-assisted intervention, amyloid-beta, tau, neuroinflammation, adult hippocampal neurogenesis

## Abstract

Alzheimer’s disease (AD) is an irreversible neurodegenerative brain disorder with aggregation of amyloid-beta (Aβ) and tau as the pathological hallmarks. AD is the most common form of dementia and is characterized by a progressive decline of cognition. The failure of pharmacological approaches to treat AD has resulted in an increased focus on non-pharmacological interventions that can mitigate cognitive decline and delay disease progression in patients with AD. Animal-assisted intervention (AAI), a non-pharmacological intervention, improves emotional, social, and cognitive dysfunction in patients with neurodegenerative diseases. In particular, AAI is reported to mitigate the effects of cognitive impairment in patients with AD. Despite the positive effects of AAI on cognitive dysfunction in patients with AD, there have been no studies on how AAI affects AD-related pathologies. This review postulates potential neurological mechanisms of emotional or social interaction through AAI in countering AD-related pathologies, such as Aβ deposition, tau hyperphosphorylation, neuroinflammation, and impaired adult hippocampal neurogenesis (AHN), and proposes insights for future research by organizing accumulated previous evidence.

## Introduction

Dementia is a chronic and progressive disease characterized by the deterioration of cognitive function (Arvanitakis et al., 2019). The WHO reported that approximately 50 million people worldwide have dementia, with 10 million incident cases every year (World Health Organization [WHO], 2020). The total number of patients with dementia is predicted to reach 82 million in 2030 and 152 million in 2050. Alzheimer’s disease (AD) is the most prevalent type of dementia, accounting for 60–70% of cases. The principal pathological characteristics of AD are amyloid-beta (Aβ) plaques aggregated by Aβ peptides and neurofibrillary tangles produced by hyperphosphorylation of tau proteins (Querfurth and LaFerla, 2010). Deposition of Aβ and tau in early AD contributes to increased neuroinflammation and neuronal loss (Mandrekar-Colucci and Landreth, 2010; Tai et al., 2015; Terada et al., 2019). In addition, abnormally accumulated Aβ and tau proteins induce dysfunction of neurotransmitter, such as glutamatergic and gamma aminobutyric acid (GABA)-ergic transmitters, leading to impairment of adult hippocampal neurogenesis (AHN) (Sun et al., 2009; Moon et al., 2014; Zheng et al., 2020). Numerous drugs have been developed to treat AD, but most drugs have failed to attenuate cognitive decline or have not slowed down the progression of the disease (Anderson et al., 2017; Cummings et al., 2020). Therefore, non-pharmacological interventions are receiving a lot of attention as an alternative treatment that can alleviate cognitive decline and influence a broad range of AD pathology.

Animal-assisted intervention (AAI), a non-pharmacological method, is a complementary goal-oriented technique that mainly results from human and animal interactions (Laun, 2003; Macauley, 2006; Sockalingam et al., 2008). The purpose of AAI is related to enhancing the emotional, social, and cognitive functions of the human. Interaction with animals provides unique communication, social support, and love-bonding for patients, which can provide a variety of mental and social health benefits to humans (Barker and Wolen, 2008; Straub, 2011). A therapeutic role of animal interactions has been reported in patients with a variety of diseases, especially neurodegenerative diseases (Laun, 2003; Macauley, 2006; Sockalingam et al., 2008; Olsen et al., 2016a,b; Bunketorp-Käll et al., 2017). Many meta-analysis studies reported that AAI has a therapeutic effect on various diseases regardless of age (Hediger et al., 2021). In patients with psychiatric disorders, such as depression, AAI has been shown to alleviate depression symptoms and restore social functions (Souter and Miller, 2007; Virués-Ortega et al., 2012). In addition, AAI relieved trauma memory in post-traumatic stress disorder patients (Germain et al., 2018; Hediger et al., 2021), and it has been reported that AAI relieves pain and improves gross motor function in children with disabilities (Charry-Sánchez et al., 2018; Zhang et al., 2021). In particular, a study has reported that AAI mitigates negative emotions, such as loneliness, anxiety, fear, and sadness in patients with AD (Mossello et al., 2011). In addition, various studies have reported that AAI affects depression, anxiety, quality of life, and cognitive function in patients with dementia (Majic et al., 2013; Kamioka et al., 2014; Olsen et al., 2016a,b). Furthermore, patients with AD who received AAI based on the reality orientation therapy protocol had significantly improved Geriatric Depression Scale and Mini-Mental State Examination scores than those who received only reality orientation therapy (Menna et al., 2016; Santaniello et al., 2020). The previous meta-analysis also showed that AAI has an effect on relieving depression in patients with dementia (Park et al., 2020). Another AAI-related meta-analysis study conducted in patients with dementia has reported that AAI may be effective in diminishing behavioral and psychological symptoms of dementia (BPSD), both in the short and long terms (Hu et al., 2018).

Despite the accumulation of evidence that supports the benefits of AAI on symptoms of AD, which includes improving cognitive impairment, BPSD, and negative emotions (Kanamori et al., 2001; Bono et al., 2015; Menna et al., 2016; Borges de Araujo et al., 2019; Santaniello et al., 2020), the underlying neurological mechanisms of these benefits have not been identified yet. In particular, we speculated that increased emotional or social interaction through AAI plays an important role in relieving symptoms in patients with AD. Thus, in this review, we focus on the increased emotional or social interactions that are increased via AAI, suggesting several possible neurological mechanisms of AAI that influence AD-related pathologies, such as Aβ deposition, tau hyperphosphorylation, neuroinflammation, and AHN disorders. Moreover, we propose future studies on AAI in AD-related pathologies.

## Hypotheses of the Neurological Mechanisms Underlying AAI in AD-Related Pathology

### The Possible Mechanisms of AAI in AD-Related Aβ Deposition and Tau Hyperphosphorylation

Regardless of any form of interaction, whether human or animal, an interruption in the interaction can negatively impact a patient experiencing loneliness and anxiety caused by a disease (Leigh-Hunt et al., 2017). Surprisingly, Aβ deposition and tau hyperphosphorylation in the brain are promoted and exacerbated by perceived social isolation or loneliness. One study reported that Aβ accumulation was observed in the brains of elderly patients who felt lonely, regardless of their diagnosis of dementia (Donovan et al., 2016). Moreover, several studies reported that amyloid precursor protein (APP)/presenilin 1 (PS1) transgenic mice in social isolation compared with standard housing have significantly increased levels of Aβ_42_ and Aβ_40_ caused by increasing γ-secretase activity and decreasing neprilysin expression (Huang et al., 2011, 2015; Hsiao et al., 2012). Similarly, some studies using rat models have reported that social isolation increases the level of glycogen synthase kinase-3β (GSK-3β) and tau hyperphosphorylation but decreases the level of Ser9-phosphorylated GSK-3β (Ren et al., 2015; Gong and Ren, 2016). This evidence suggests that social isolation and perceived loneliness can promote Aβ production and tau hyperphosphorylation in the brains of those with early AD. In addition, stress from social isolation and loneliness can also affect Aβ accumulation and tau hyperphosphorylation. Stress-induced increases in glucocorticoid levels lead to exacerbation of Aβ and tau pathologies (Green et al., 2006). In particular, patients with AD and mild cognitive impairment showed significantly increased plasma and cerebrospinal fluid (CSF) cortisol levels than patients with normal cognition and also showed dysregulation of the hypothalamic-pituitary-adrenal axis (Popp et al., 2015; Ouanes and Popp, 2019). Increased levels of glucocorticoids induced by stress in 3xTg mice accelerate the development of neurofibrillary tangles by increasing the accumulation of somatodendritic tau and raising the levels of intraneuronal Aβ through increased levels of APP and β-secretase (Green et al., 2006). Interestingly, short-term interactions between dogs and owners reduced cortisol levels in their owners (Handlin et al., 2011). In addition, participants who directly touched dogs or cats through animal visiting programs and had sensory interactions with animals showed decreased cortisol levels compared with other groups that did not interact with animals (Pendry and Vandagriff, 2019); however, as these mechanisms were not confirmed by directly applying AAI to animal models or patients with AD, future studies are required for confirming the direct effect of AAI on Aβ deposition and tau hyperphosphorylation using positron emission tomography in preclinical or patients with prodromal AD.

### The Possible Mechanisms of AAI in AD-Related Neuroinflammation

A variety of stresses, including a decrease in emotional or social interactions, regardless of the type of interaction, have been reported to increase the levels of inflammatory cytokines and the activity of the inflammatory immune system (Coussons-Read et al., 2007; Audet et al., 2014). In brains of patients with AD, the innate immune response induced by various stresses is converted into chronic inflammation, which contributes to neurodegeneration (Heneka et al., 2015). Chronic stress in the AD brain promotes the proliferation of glial cells and increases secretion of stress-induced cytokines, such as interleukin (IL)-1β, IL-6, IL-12, IL-18, and tumor necrosis factor-alpha (TNF-α), resulting in impaired neurogenesis and synaptic plasticity (Ricci et al., 2012). Neurodegeneration also contributes to neuroinflammation, the development of a vicious cycle between neuroinflammation and neurodegeneration, and the acceleration of progression of AD and cognitive decline (Kempuraj et al., 2016). In particular, the disconnection of social interaction increases the levels of pro-inflammatory cytokines, such as IL-6 and TNF-α, in the anterior insula and dorsal anterior cingulate cortex (Slavich et al., 2010). In addition, stress due to the disconnection of social interaction increases microglia and astrocyte activity in the hippocampus of aged APP/PS1 mice (Huang et al., 2015). Surprisingly, a meta-analysis of the association between social support-social integration and neuroinflammation has shown that inflammatory cytokines were significantly reduced in the social support group (Uchino et al., 2018). In addition, social interaction significantly reduced the activity of IL-1β and TNF-α in a rat model with AD (Ali et al., 2017). These results reveal that increased emotional or social interaction alleviates the neuroinflammatory response. Importantly, AAI remarkably reduced Stress Visual Analog Scale scores in older patients in intensive care units and slightly reduced IL-1β levels in saliva (Branson et al., 2020). Taken together, increased emotional or social interaction through AAI may mitigate AD-related neuroinflammation by reducing stress-induced pro-inflammatory cytokine release and the activity of microglia and astrocytes. Future studies are needed to confirm the direct effects of AAI on AD-related neuroinflammation by detecting cytokine levels in saliva, blood, and CSF in patients with prodromal or mild AD.

### The Possible Mechanisms of AAI in AD-Related Neurodegeneration

Decreased emotional or social interactions robustly contribute to below-normal-range cognitive function and more rapid cognitive impairment in the development of AD (Wilson et al., 2007). In a study examining the levels of neurotransmitter immunoreactive neurons using immunohistochemistry, social isolation reduced cholinergic neurons of the vertical diagonal bands in wild-type mice (Huang et al., 2011). In addition, an environment with reduced interaction decreased hippocampal volume, synaptophysin expression, and myelin basic protein expression in aged APP/PS1 mice (Huang et al., 2015). The cholinergic neurons of the vertical and horizontal diagonal bands of Broca’s area, noradrenergic neurons of the locus coeruleus, and serotonergic neurons of the raphe nucleus were significantly reduced in isolated APP/PS1 mice compared with APP/PS1 standard housing controls (Huang et al., 2011). Surprisingly, according to various animal models and human studies, the opioid system is a major mediator that influences social functions including social bonding (Barr et al., 2008; Copeland et al., 2011; Trezza et al., 2011; Colonnello et al., 2017). Particularly, opioid receptors are associated with the regulation of neurotransmitters, such as glutamate, GABA, acetylcholine, serotonin, and noradrenaline. In addition, a study investigating the levels of NR2A or NR2B, which is involved in the dendritic arbor and neuronal development in opioid receptor systems, reported that the expression of receptors significantly decreased in both healthy and APP/PS1 mice living in an environment with reduced social interaction compared with a standard housing group (Huang et al., 2011). These results suggest that decreased emotional or social interactions may exacerbate neurodegeneration by inducing dysfunction of the opioid system in AD. Another study using functional magnetic resonance imaging (fMRI) showed that rearing pet insects positively affected executive functions through the activity of the right dorsal lateral prefrontal cortex and parietal cortex in elderly women (Park et al., 2019). In addition, the increased interaction through AAI in a study that measured changes in oxygenated and deoxygenated hemoglobin concentrations using near-infrared spectroscopy significantly increased the oxygenated hemoglobin concentration in the prefrontal cortex of mood disorders patients with low prefrontal cortex activity (Aoki et al., 2012). These results suggest that AAI may induce biological and physiological changes in the prefrontal cortex. Furthermore, application of the combination of AAI and cognitive rehabilitation techniques improved executive function, social skills, and mood regulation in patients with acquired brain injury (Stapleton, 2016; Gocheva et al., 2018), and improved cognitive function and depressive status in patients with AD (Santaniello et al., 2020). This evidence implicates direct effects of increased interaction through AAI on the brain, and that AAI may also improve functional connectivity and activity in the brains of patients with AD. Therefore, increased emotional or social interaction through AAI might contribute to the inhibition of AD-related neurodegeneration by suppressing the loss of various neurotransmitter-releasing neurons and improving synaptic plasticity by mitigating the dysfunction of the opioid system in the brains of patients with AD. However, because there is little evidence about the mechanism of AAI in the brain of patients with AD, future studies are required for confirming the direct effect of AAI on alterations of networks and circuits by using fMRI or diffusion tensor imaging in patients with prodromal or mild AD.

### The Possible Mechanisms of AAI in AD-Related Impaired AHN

The subgranular zone (SGZ) of the hippocampal dentate gyrus (DG) exhibits a unique phenomenon of the creation of new neurons and glial cells throughout life in adult mammals. This phenomenon is called AHN (Ming and Song, 2011). The AHN process is divided into proliferation, differentiation, maturation, and integration. In addition, this process provides an incomparable degree of neuronal plasticity to the entire hippocampal circuitry. Therefore, AHN plays a significant role in network maintenance and structural plasticity in the hippocampus (Mu and Gage, 2011). Interestingly, several brain disorders, such as neurodegenerative diseases and mood disorders, exhibit dysfunction and dysregulation of AHN, and some symptoms of these disorders can be partially elucidated by impaired AHN (Toda et al., 2019). In addition, many studies have indicated that AHN is significantly reduced in patients with AD (Moreno-Jimenez et al., 2019) and various mouse models with AD (Rodriguez et al., 2008; Faure et al., 2011; Moon et al., 2014; Wirths, 2017). Social stress, such as decreased emotional or social interactions, is associated with AHN impairment. A study investigating the effects of decreased emotional or social interactions on neurogenesis in young marmosets using BrdU, a cell proliferation marker, reported that social deprivation has induced stress and reduced hippocampal neurogenesis (Cinini et al., 2014). In addition, many studies using mouse models with AD have reported that decreased emotional or social interactions exacerbate AD-related pathologies and cognitive impairment (Pietropaolo et al., 2009; Huang et al., 2011, 2015); however, the application of social interactions in an mouse model with AD decreased histone deacetylase 2 (HDAC2) expression and occupancy of HDAC2 in the promoter region of brain-derived neurotrophic factor (BDNF) exon IV, resulting in upregulated BDNF expression in the hippocampus region through increased acetylation of H3K9 and H4K12 histones. Increased BDNF expression improves AHN, synaptic density, and cognition (Hsiao et al., 2014, 2018). These results suggest that increased emotional or social interaction through AAI might involve epigenetic mechanisms and can contribute to cognitive improvement by suppressing the impairment of AHN in patients with AD. Unfortunately, there is no direct evidence of influence of AAI on epigenetic regulation. Therefore, further studies are needed on the epigenetic mechanisms of emotional or social interaction through AAI in patients with AD.

## Discussion

The physiological changes that accompany psychological distress and social isolation decrease state of health of a person, accelerate the development and progression of chronic diseases, and increase mortality (McEwen, 1998). Surprisingly, positive interactions involving companion animals have been found to affect the psychosocial status and reduce psychosocial distress and stress responses. In particular, interactions with animals seem to reduce psychosocial distress by making people appear kinder in a variety of situations (Friedmann and Lockwood, 1991; Rossbach and Wilson, 1992). Interestingly, situations with animals are perceived as more familiar, comfortable, reciprocal, valuable, safe, and happy than situations without animals. In particular, AAI, which is an intervention that uses animals to treat various diseases, has a beneficial effect on psychological and emotional symptoms (Churchill et al., 1999; White et al., 2020). Unfortunately, although various factors, such as the AAI method, applied animal, frequency of AAI, and subject of AAI, are important when evaluating the effectiveness of AAI, these factors differ from study to study. These variables make it difficult to integrate and standardize studies on AAI. Despite these barriers, animals are widely used for the emotional and psychological treatment of various patients (Sable, 1995; Friedmann and Son, 2009; Amerine and Hubbard, 2016). Therefore, this review outlined the neurological mechanisms of AAI, which are putatively focused on the familiar interactions between animals and humans, especially emotional and social interactions, regardless of the animal type used in AAI.

Animal-assisted intervention has therapeutic effects in a variety of health areas. AAI has a beneficial effect on behavioral areas through interaction with animals and the elderly, such as seeing, talking, and touching animals as well as interactions with elderly people talking about animals (Nordgren and Engstrom, 2014). In addition, taking care of animals promotes a sense of motivation for performance in the work and increases self-esteem in older adults, thus showing positive effects in psychosocial areas (Menna et al., 2016). In physical areas, AAI provides improvement of muscle strength and a sense of balance (Grubbs et al., 2016). Furthermore, AAI relaxes the cardiovascular stress response in the elderly (Robinson et al., 2015). In AD, AAI is known to be effective in not only relieving emotional stability and depression through love-bonding between animals and patients with AD but also increasing physical exercise and promoting motivation through walking with and touching animals (Tribet et al., 2008; Klimova et al., 2019). In addition, sundowning, which is one of the symptoms in AD, is also reduced by relieving anxiety through AAI (Churchill et al., 1999). Surprisingly, animals can estimate the risk of patients with AD, and they can prevent them from the risks faced by patients with AD through barking or non-verbal behavior. Unfortunately, in the case of AAI, the patients and animals participating need special care to prevent animal infectious diseases, irritability, and injuries during visits (Jofre, 2005). In particular, AAI should always be performed according to the recommended guidelines of a structured program, trying to prevent infection with animal-related diseases, and applying appropriate interaction models for disease treatment. Interestingly, in a study of risk analysis for the health of people who interact with animals, it was observed that interactions with animals under controlled environmental conditions are beneficial to their health (Brodie et al., 2002; DiSalvo et al., 2006). It is important that the application of AAI be carried out in a controlled environment. Furthermore, an AAI program should be developed through the interaction of various AAI specialists, such as physical therapists, neurologists, psychiatrists, veterinary public health professionals, psychologists, and occupational therapists, and carried out in accordance with the recommended guidelines (Cevizci et al., 2013). An AAI program, created and carried out through a multidisciplinary approach of various specialists, is a professional approach with a clear therapeutic effect among various non-pharmacological interventions.

The multi-targeting character of non-pharmacological interventions is attracting attention as a novel and innovative treatment strategy that exhibits therapeutic effects in various diseases (Olazaran et al., 2010). Unfortunately, the multi-targeting characteristic is considered unscientific, as, it is difficult to determine the exact therapeutic effect of the single non-pharmacological intervention. It is also difficult to convert and apply the intervention into a preclinical experimental study, as the understanding of the mechanism of action is insufficient. To overcome disadvantages and to use non-pharmacological intervention for the treatment and prevention of dementia or other neurodegenerative diseases, scientific evidence-based studies, such as reviews of neurological and molecular mechanisms and preclinical experimental studies, are necessary. Similarly, even in the case of AAI, preclinical experimental study methods are required to explain the molecular and neurological mechanisms for various effects because it remains difficult for experimental animals to establish the concept of animals from the standpoint of companion animals or experimental animals. In addition, many studies that have applied AAI to patients with AD have only investigated the ability to improve AD-related symptoms, such as cognitive dysfunction, depression, anxiety, motivation, and exercise. Because studies confirming neurological/pathologic changes, including changes in the inflammatory cytokines, Aβ, tau level, and neural circuits in the central nervous system, are insufficient, it is difficult to show the mechanisms by which AAI alleviates AD-related pathologies; however, in this review, we highlighted the value of AAI as a non-pharmacological intervention through the hypothesis that AAI may also affect AD-related pathologies and presented insights for future studies.

**FIGURE 1:**
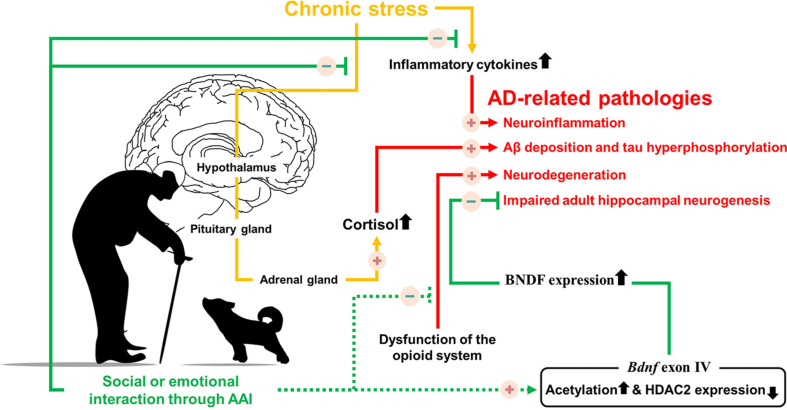
Possible neurological mechanism involved in Alzheimer’s disease symptoms alleviation by providing emotional or social interaction through animal assisted intervention. Solid line indicates the known mechanisms, dotted line indicates hypothesized mechanisms.

The positive-constructive bond resulting from human-animal interactions is the key point for initiating the beneficial effects of AAI. These effects are known to regulate stress, hormone imbalances, cognitive dysfunction, and various symptoms (Table 1). Despite the lack of evidence that AAI can alleviate AD-related pathologies, we hypothesized that emotional or social interactions through AAI could rescue cognitive impairment by alleviating AD-related pathologies. Therefore, based on the accumulated evidence, we speculated on the neurological mechanisms by which AAI affects AD-related pathologies, such as Aβ deposition, tau hyperphosphorylation, neuroinflammation, and impaired AHN (Figure 1). This review confirms the value of AAI as a non-pharmacological intervention through the hypothesis that AAI used for symptomatic relief of AD may also affect AD-related pathologies and suggests insights for future research.

**TABLE 1:** The effects of AAI on healthy individuals and patients with neurodegenerative disease.

	Participants (Experimental/Control)		Intervention		Main results	References
		
Functional decline	Age	Sample size	Type of AAI	Period/total number of sessions		
Hormone imbalance	42 ± 8/53 ± 10	10/10	Short-term interaction between dogs and their owners	1 session	Oxytocin levels ↑	Handlin et al., 2011
					Cortisol levels ↓	
					Insulin levels ↓	
	19.94 ± 1.66/−	249/−	Animal visitation programs	1 session	Cortisol levels ↓	Pendry and Vandagriff, 2019
Stress	39.9 ± 16.2/−	9/−	Animal-assisted therapy	5 sessions	Stress ↓	González-Ramírez et al., 2013
	18–25/−	68/−	Animal-assisted programs	–	Stress ↓	Haggerty and Mueller, 2017
	60–86	20/20	Animal-assisted activity	20 sessions	Stress ↓	Branson et al., 2020
Cognitive dysfunction	75.25 ± 6.06/75.1 ± 5.83	16/7	Animal-assisted therapy	6 months/24 sessions	MMSE ↑	Menna et al., 2016
	76.6 ± 5.0/75.0 ± 6.3	65/31	Animal-assisted therapy	6 months/24 sessions	MMSE ↑	Santaniello et al., 2020
	78.6 ± 7.4/−	9/−	Equine-assisted therapy	10 weeks/20 sessions	MMSE ↑	Borges de Araujo et al., 2019
	79.43 ± 6.06/83.4 ± 7.22	7/20	Animal-assisted therapy	11 weeks/5 sessions	MMSE ↑	Kanamori et al., 2001
	82.1 ± 6.2/78.3 ± 10.3	12/12	Animal-assisted therapy	8 months/16 sessions	MMSE ↑	Bono et al., 2015
Various symptoms	59–67	3/−	Animal-assisted therapy	12 sessions	Aphasia ↓	Macauley, 2006
	65 or older	22/26	Animal-assisted therapy	12 weeks/24 sessions	Balance ↑	Olsen et al., 2016a
	65 or older	25/26	Animal-assisted therapy	12 weeks/24 sessions	Quality of Life ↑	Olsen et al., 2016b
					Depression ↓	
	62.6 ± 6.5/63.7 ± 6.7	41/41	Horse-riding therapy	12 weeks/6 sessions	Stroke recovery ↑	Bunketorp-Käll et al., 2017
					Balance ↑	
					Balance ↑	
	81.33 ± 10.20/82.07 ± 8.65	27/27	Animal-assisted therapy	10 weeks/10 sessions	Agitation ↓	Majic et al., 2013
					Depression ↓	
	79 ± 6/−	10/−	Animal-assisted activity	1 week/3 sessions	Neuropsychiatric symptom ↓	Mossello et al., 2011
					Sadness ↓	
					Pleasure ↑	
					Alertness ↑	
	75.25 ± 6.06/75.1 ± 5.83	16/7	Animal-assisted therapy	24 weeks/24 sessions	Cognitive function ↑	Menna et al., 2016
					Depression↓	

## Author Contributions

SK, YN, M-JH, CP, MM, and D-HY wrote this review article. All authors contributed to the article and approved the submitted version.

## Conflict of Interest

The authors declare that the research was conducted in the absence of any commercial or financial relationships that could be construed as a potential conflict of interest.
